# Reduced evolutionary rates in HIV-1 reveal extensive latency periods among replicating lineages

**DOI:** 10.1186/s12977-014-0081-0

**Published:** 2014-10-16

**Authors:** Taina T Immonen, Thomas Leitner

**Affiliations:** Theoretical Biology & Biophysics, Los Alamos National Laboratory, Los Alamos, NM 87545 USA

**Keywords:** HIV-1 latency, Phylogenetics, Molecular clock, False discovery rate, Dynamic modeling

## Abstract

**Background:**

HIV-1 can persist for the duration of a patient’s life due in part to its ability to hide from the immune system, and from antiretroviral drugs, in long-lived latent reservoirs. Latent forms of HIV-1 may also be disproportionally involved in transmission. Thus, it is important to detect and quantify latency in the HIV-1 life cycle.

**Results:**

We developed a novel molecular clock–based phylogenetic tool to investigate the prevalence of HIV-1 lineages that have experienced latency. The method removes alternative sources that may affect evolutionary rates, such as hypermutation, recombination, and selection, to reveal the contribution of generation-time effects caused by latency. Our method was able to recover latent lineages with high specificity and sensitivity, and low false discovery rates, even on relatively short branches on simulated phylogenies. Applying the tool to HIV-1 sequences from 26 patients, we show that the majority of phylogenetic lineages have been affected by generation-time effects in every patient type, whether untreated, elite controller, or under effective or failing treatment. Furthermore, we discovered extensive effects of latency in sequence data (*gag*, *pol,* and *env*) from reservoirs as well as in the replicating plasma population. To better understand our phylogenetic findings, we developed a dynamic model of virus-host interactions to investigate the proportion of lineages in the actively replicating population that have ever been latent. Assuming neutral evolution, our dynamic modeling showed that under most parameter conditions, it is possible for a few activated latent viruses to propagate so that in time, most HIV-1 lineages will have been latent at some time in their past.

**Conclusions:**

These results suggest that cycling in and out of latency plays a major role in the evolution of HIV-1. Thus, no aspect of HIV-1 evolution can be fully understood without considering latency - including treatment, drug resistance, immune evasion, transmission, and pathogenesis.

**Electronic supplementary material:**

The online version of this article (doi:10.1186/s12977-014-0081-0) contains supplementary material, which is available to authorized users.

## Background

HIV-1 hidden in latent reservoirs is a major obstacle in eradicating the virus, as the reservoirs may be very long-lived [1-4]. Discontinuing antiretroviral treatment (ART) generally results in rebound of viremia due to the activation of latent virus [5]. Thus, the eradication of HIV-1 necessarily involves reversing latency, and preventing the activated virus from establishing new rounds of infection under ART [6]. If the latent reservoir(s) could be eradicated, ART would kill the replicating HIV-1 and cure the patient [7,8]. Hence, accurately quantifying the size of the latent reservoir is vital for guiding potential eradication efforts and, if eradication is possible, determining when the latent reservoir is exhausted.

Recent findings suggest that latency may also be involved in transmission, as latent forms appear to be preferentially transmitted than more recent variants [9-12]. This implies that activated latent viruses are able to survive and propagate under immune pressure to reach sufficient numbers in the plasma to be transmitted.

Because HIV-1 accumulates mutations while replicating, but not when latent, a virus lineage that has been latent for some time will show a slower effective rate of genetic evolution. Thus, latency effectively alters the rate of evolution of the virus, yielding multiple molecular clock rates in a patient.

To detect latency in HIV-1 DNA sequence data, we developed a phylogenetic method that identifies lineages that have evolved less than expected under a single mutation rate. Hence, we based our approach on an explicit mechanistic hypothesis that explains overdispersion, i.e. rate variation larger than expected from a single Poisson process, rather than using a statistical distribution with wider variance as has been done in generic approaches using relaxed clocks [13].

In addition to the generation-time effect caused by latency, hypermutation, recombination, and selection may alter apparent evolutionary rates. Both positive and negative selection may affect divergence and diversity [14,15], while activation and reintroduction of latent forms into the replicating population may expand diversity only on the lower tail beyond what could be described by a single Poisson process. Our method addresses each of these effects to reveal the contribution of latency to the evolution of HIV-1.

We first tested our latency detection method on simulated phylogenies in which randomly selected branches were affected by latency. We then applied our method to 26 data sets, consisting of HIV-1 DNA sequence alignments derived from the plasma and latent reservoirs of patients that were either untreated, successfully treated, had experienced treatment failure or were elite controllers [16-19]. To understand our phylogenetic findings from a theoretical perspective, we developed a dynamic model of virus-host interactions under neutral evolution, and investigated the proportion of lineages that have ever experienced latency. Both our phylogenetic and modeling results suggest that latency affects the majority of phylogenetic lineages in most patients.

## Results

### Correct identification of latent lineages in simulated data

We developed a method to identify all lineages in a phylogeny that have evolved significantly less than at least one other lineage, assuming that HIV mutates according to a Poisson process with a single molecular clock rate when replicating, and does not mutate when latent.

**Figure 1:**
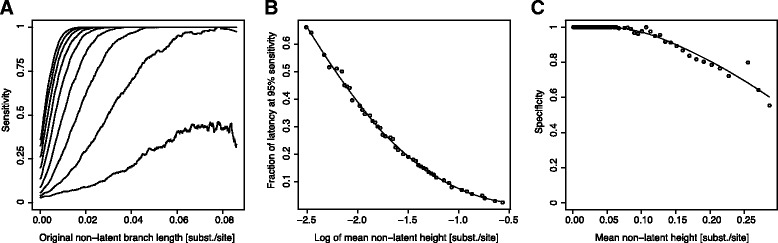
**Success of phylogenetic latency detection in critical simulation scenarios.** Detection of latency-affected lineages was done by pair-wise comparisons of all taxa, testing whether the shorter lineage was significantly shorter according to a Poisson test. **(A)** Sensitivity results from simulations with 100 taxa and one random branch affected by latency at *f*_latent_ = 0.1–0.9 of the corresponding original non-latent genetic distance. Lines show moving average for general trends (*f*_latent_ = 0.9–0.1, left to right). Individual simulation results are shown in the *Supplement* (Additional file 1: Figure S2). **(B)** The minimum latency fraction on an affected branch to achieve 95% sensitivity as a function of the mean height of non-latent taxa from the MRCA of all taxa. The height is in log10-units to facilitate reading short tree height performance. **(C)** Specificity as a function of the mean height of non-latent taxa from the MRCA of all taxa. Panels B and C have loess curves fitted to show general trends.

To explore the limitations of our method, we tested its performance in critical simulations. Figure 1A shows simulation results from random phylogenies with 100 taxa each, where one random branch was latent for a fraction of its evolutionary time (*f*_latent_). Note that in each simulated phylogeny, the length and position of the latent branch may differ, and thus also the number of taxa affected by latency (range 1—(*N*-1) latent taxa). Overall, we were able to recover the lineages affected by latency even when *f*_latent_ approached small values; for branches >0.02 subst/site and *f*_latent_ > 0.4 we observed >90% sensitivity (Figure 1A). Because estimating the mean height of non-latent taxa from the MRCA of all taxa per time point is easy in real data, we also simulated trees varying the tree height and identifying the minimum proportion of latency in lineages that were detected as latent to achieve 95% sensitivity (Figure 1B). This means that, for instance, if the mean non-latent height in a sample was 0.1 substitutions/site, we can detect at 95% sensitivity latency periods as short as 7% of the non-latent genetic distance, while in a population with small divergence of 0.01 substitutions/site height, the affected lineages must have been latent for at least 60% of the evolutionary time. Clearly, while it is easy to detect latency on longer branches, the effect of latency on short branches is small and therefore also difficult to detect. The specificity of our method was at an overall 98% for genetic distances at 0.1 substitutions/site, and decreased slowly to approximately 70% at 0.25 substitutions/site (Figure 1C), a distance one rarely detects in a within-patient single time point sample (Additional file 1:Table S2).

**Figure 2:**
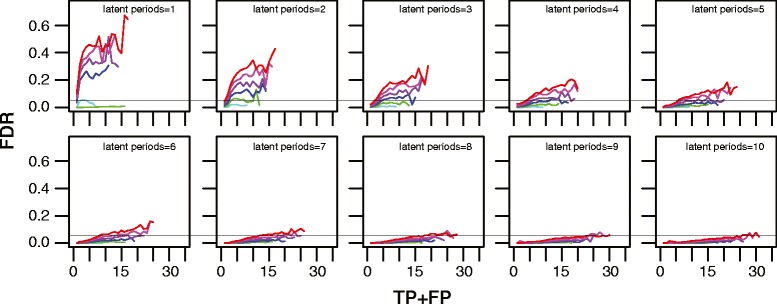
**The false discovery rate (FDR) of our phylogenetic latency detection method.** Because our latency detection method performs many tests, the risk of false positives increases. This figure shows the FDR as a function of the total number of positives detected (TP + FP). The FDR depended strongly on the number of latency periods (panels), and on the number of taxa compared (lines); cyan = 10 taxa, green = 20 taxa, blue = 30 taxa, purple = 40 taxa, magenta = 50 taxa, and red = 60 taxa. Each line shows the mean of 50,000 simulated random phylogenies at *f*_latent_ = 0.5–0.9. Means were calculated when ≥50 observations occurred at any TP + FP level. The hairline shows FDR = 0.05.

As our method performs many tests, the risk of false positives increases. While many multiple test correction procedures have been developed, e.g., [20-22], none are appropriate for our situation where tests are phylogenetically correlated and one-sided. Thus, we explored by simulation the behavior of the false discovery rate (FDR = FP/[TP + FP]). Figure 2 shows simulation results from 3 million random phylogenies; the FDR had a strong inverse relationship to the number of latent periods, and was directly proportional to both the number of taxa and the number of detected positives (TP + FP). The FDR was, however, only modestly affected by *f*_latent_. When the number of taxa was small (10–20 per time point), the FDR was <5% regardless of the number of detected positives. As the number of latent periods increased, the number of taxa and the number of detected positives had less effect. At 5 or more latency periods the FDR was typically <10%, and at 8 or more latency periods the FDR was generally <5% (hairline in Figure 2).

### Reduced evolutionary rates identify latency in the majority of lineages

Next, we applied our phylogenetic latency detection method to real data from 26 longitudinally sampled patients, including both HIV-1 sequences from the actively replicating plasma population and latently infected resting CD4+ T cells [16-19]. Because reduced evolutionary rates may be the result of other processes in addition to latency (which causes generation-time effects), i.e., artificially long reference taxa due to hypermutation or recombination, or selection making some lineages evolve faster or slower than others, we first removed hypermutants and potential recombinants that could bias our results (Table 1). To remove the potential bias that might cause rate differences due to selection, we analyzed phylogenies based on 3^rd^ codon positions only. First, the results based on 3^rd^ codon positions were very similar to the results based on all positions (compare Table 1 and Additional file 1: Table S1). Second, as we detected slightly more recombinants and fewer latents in all positions, this suggests that some potential recombinants that we identified based on all positions probably were in fact misidentified selection effects. Note that it is well known that selection and recombination are confounding factors difficult to discriminate [23-25]. As our goal here was to remove both selection and recombination effects in order to see the effects of latency, however, it did not matter in which way we removed them.

**Table 1:** **Summary of patient data and latency results based on 3** ^**rd**^ **codon positions**

**Patient ID**	**Patient type**	**Gene**	**L**	**Time points**	**No. taxa**	**H**	**R**	**No. (%) latent taxa detected**			
								**Total**	**Plasma**	**Reservoir**	**FP**
K-B	U	*env*	714	3	49	1	0	43 (87.8)	24 (85.7)	19 (90.5)	0.7
K-G	U	*env*	729	3	49	0	0	38 (77.6)	18 (62.1)	20 (100)	1.0
O-1	E	*gag*	1386	3	20	0	0	16 (80.0)	2 (40.0)	14 (93.3)	0.8
O-7	E	*gag*	1271	9	53	1	3	27 (50.9)	26 (55.3)	1 (16.7)	0.8
O-8	E	*gag*	1298	7	101	0	0	60 (59.4)	15 (27.8)	45 (95.7)	4.7
O-9	E	*gag*	1271	5	50	2	0	32 (64.0)	3 (15.0)	29 (97.0)	0.6
M-1	F	*pol*	985	1	22	0	0	12 (54.5)	6 (60.0)	6 (50.0)	0.1
M-96	F	*pol*	637	2	19	0	0	0 (0.0)	0 (0.0)	0 (0.0)	0
M-100	F	*pol*	637	1	16	0	0	4 (25.0)	2 (40.0)	2 (18.2)	0.3
M-102	F	*pol*	637	3	108	0	1	59 (54.6)	12 (42.8)	47 (58.8)	0.9
M-105	F	*pol*	637	3	54	0	0	48 (87.3)	24 (82.8)	24 (92.3)	0.1
M-110	F	*pol*	637	1	12	0	0	11 (91.7)	5 (83.3)	6 (100)	0.1
M-112	F	*pol*	637	1	25	0	0	18 (72.0)	5 (55.6)	13 (81.3)	0.1
M-122	F	*pol*	637	1	14	0	0	7 (50.0)	0 (0.0)	7 (87.5)	0.7
M-126	F	*pol*	637	1	18	0	0	5 (27.8)	4 (66.7)	1 (0.08)	0.5
M-127	F	*pol*	985	1	14	0	0	8 (57.1)	4 (100)	4 (40.0)	0.1
M-137	F	*pol*	637	2	33	0	0	17 (51.5)	1 (0.06)	16 (94.1)	0.1
M-138	F	*pol*	985	1	11	0	0	0 (0.0)	0 (0.0)	0 (0.0)	0
B-113	T	*pol*	546	10	47	0	0	33 (70.2)	11 (47.8)	22 (91.7)	0.1
B-134	T	*pol*	546	5	76	0	0	54 (71.1)	1 (14.3)	53 (76.8)	1.6
B-136	T	*pol*	546	10	110	0	0	50 (45.5)	5 (11.4)	45 (68.2)	20
B-139	T	*pol*	546	10	126	0	0	88 (69.8)	18 (41.9)	70 (84.3)	4.9
B-147	T	*pol*	546	10	132	0	0	112 (84.8)	28 (65.1)	84 (94.4)	2.5
B-148	T	*pol*	546	4	152	0	0	129 (84.9)	7 (50.0)	122 (88.4)	4.5
B-154	T	*pol*	546	18	337	0	0	154 (45.7)	77 (35.2)	77 (65.3)	5.9
B-154	T	*env*	306	3	64	3	0	50 (78.1)	30 (71.4)	20 (90.9)	1.3

Footnotes: Patient type, U = untreated, E = elite controller, F = failed treatment, T = successful treatment; L = original sequence length of all codon positions; H = number of hypermutants removed; R = number of recombinants removed; No. taxa = number of taxa after H and R removal; FP = estimated number of false positives. Additional results in Additional file 1: Tables S1 and S2.

To evaluate the potential of overestimating latency due to statistical effects, we estimated the total number of FP’s per patient (Table 1) based on the inferred FDR per time point (Additional file 1: Table S2). The number of FP’s was very small in all patients (on average 2/patient), except B-136 because of a high FDR at time point 25 weeks (0.3 FP/[TP + FP]). Furthermore, to facilitate comparison of the real patient data to our phylogenetic simulations (Figure 1B), we determined the mean height of non-latent taxa from the MRCA of all taxa per time point (Additional file 1: Table S2). Typically, the within-patient height in a sample was 0.054 substitutions/site, which corresponds to correctly identifying lineages that have been latent for at least 15% of the height at a sensitivity of 95%, and nearly 100% specificity. The variance was large, however, with a standard deviation of 0.046 and range of 0.001—0.24 substitutions/site, emphasizing that there are large patient and virus specific differences in the observed evolutionary rates.

Overall, regardless of whether the patient was untreated, successfully treated, had experienced treatment failure, or was an elite controller, the generation-time effects of latency was detected in the majority of phylogenetic lineages (Table 1). The overall mean fraction of latent lineages across all patients was 59% (57% corrected for estimated FP). More specifically, we found latency in 45% of plasma-derived virus (the actively replicating population at sampling), and 68% of latent reservoirs consisting of resting CD4+ cells. Finding more lineages affected by generation-time effects in the latent reservoir confirms that our detection method picks up the relevant signal. The fact that we find a high fraction of lineages affected by generation-time effects in the plasma suggests that activated latent viruses are able to compete against more recent variants that have evolved under selection pressure from the immune response. The proportion of plasma-derived virus measurably affected by latency was 42% for treated patients, 82% for untreated patients, 35% for elite controllers, and 44% for patients with treatment failure, not differing significantly between groups (p = 0.24, Kruskal-Wallis rank sum test).

### Latency occurs throughout infection

**Figure 3:**
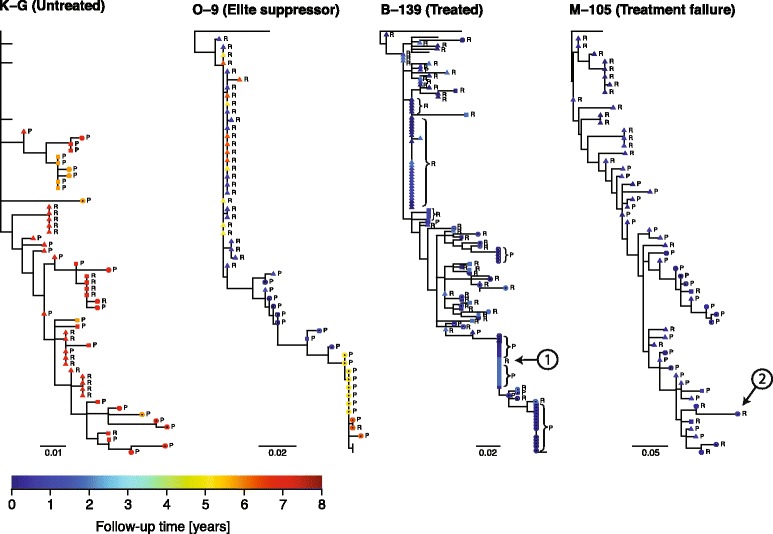
**Latency detection in HIV-1 phylogenies.** To avoid effects of selection, only 3^rd^ codon DNA positions were used for phylogenetic reconstruction and latency detection. Latency was detected in an untreated patient (K-G), an elite suppressor (O-9), a successfully treated patient (B-139), and in a patient with failed treatment (M-105). These are representative patients of each group in Table 1. All 26 patient trees are shown in the online supplementary materials (Additional file 1: Figure S1). Taxa are labeled P for plasma-derived virus and R for resting CD4+ T cell pro-virus. Filled symbols indicate significant reduction in genetic distance (circles p < 0.05; squares p < 0.01; triangles p < 0.001). Crossed circles indicate potential recombinants, omitted from latency detection. Unlabelled ingroup taxa indicate additional sequences from neither plasma nor resting cells. Color indicates time since 1^st^ sample of patient. Patient K-G was rooted at 1^st^ sample close to PHI (unlabeled taxa), and the other patients were rooted by an outgroup (HIV-1 HXB2, unlabelled). The circled numbers are observations of interest discussed in the text.

Figure 3 shows representative examples of serially sampled phylogenies based on 3^rd^ codon positions only of each patient type. All patient trees are shown in the *Supplement* (Additional file 1: Figure S1). Clearly, the majority of taxa have been affected by significant reductions in their evolutionary distances (filled symbols). The HIV-1 phylogeny from patient K-G shows that shortly after infection, the difference in how far lineages have evolved is negligible. This makes sense, as cycling through latency should have a small effect on the number of mutations early in infection; few mutations could have accumulated when not many replication cycles have occurred. In patients K-B and K-G (untreated patients), the first time point samples were used to root the trees. At later time points, in K-G and all other patients, latency had affected most lineages. All patients show continuous evolution, interrupted by clonal expansions of latency-affected taxa, where plasma derived virus are identical to reservoir viruses (e.g., circle-1 in Figure 3). Notably, while viruses derived from reservoirs have often evolved less, both reservoir and plasma viruses show effects of latency-reduced evolution in many patients.

### Dynamic model predictions agree with phylogenetic findings

**Figure 4:**
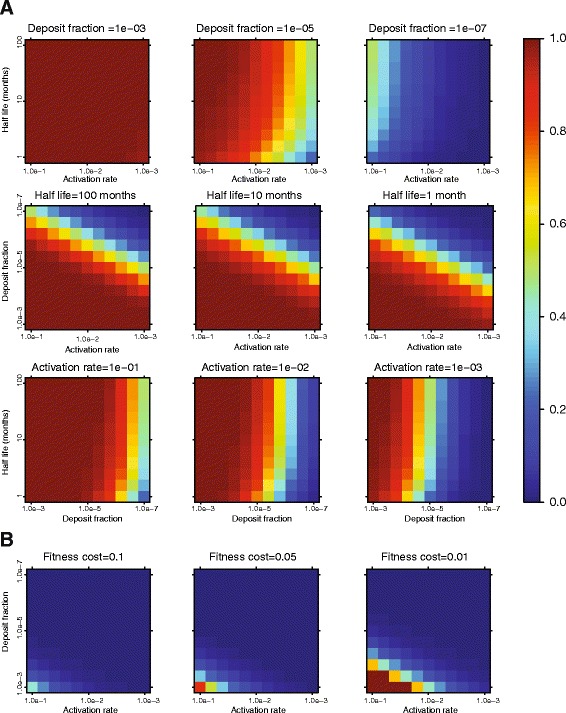
**Dynamic modeling of ever-latent HIV-1 lineages. (A)** Assuming neutral evolution most parameter regimes suggest that the vast majority of all HIV-1 lineages have been latent during their evolution. The heat map scale, blue to red, shows fraction of ever-latent (0.0-1.0) in an untreated patient after 10 years of infection within previously described parameter ranges for the fraction of HIV-1 that become deposited into the latent pool, the half-life of the latent pool, and the activation rate of latent HIV-1 back into the replicating population. **(B)** Assuming differential fitness costs for ever-latents and never latents, the fraction ever-latents surviving is reduced compared to a neutral model. The fitness plots were generated assuming a half-life of 10 months.

To independently investigate our phylogenetic findings, we expanded a previous dynamic pathogen-host model [26,27] by tracking viruses that have cycled through a latent reservoir at some time in their past (ever-latent HIV-1). The dynamic modeling suggests that under neutral evolution, the relatively small numbers of activated latent viruses will propagate in the plasma so that most HIV-1 lineages in the replicating population have been latent at some time in their past under most parameter value combinations (Figure 4A). Not surprisingly, the fraction of infections resulting in latency *η* had the largest effect on the proportion of ever-latent HIV-1 in the plasma population. At the other extreme, if latently infected cells were never activated, latency would effectively be a dead end with no effect on the plasma population. At intermediate values, however, we observed massive fractions of ever-latent virus. Interestingly, the reservoir half-life had a negligible effect on the fraction of ever-latents. Regardless of parameter values, the fraction of ever-latents grew slowly over time after initial establishment, implying that 1) latency deposition starts immediately after infection and 2) that the latent pool expands its potential to influence HIV-1 adaptation over time, both by concentration and mutational spectrum.

Assuming neutrality, however, ignores immune selection and fitness differences between ever- and never-latent viruses. Therefore, we introduce a constant fitness cost (1-ε_E_) on ever-latent lineages, making it more difficult for them to survive. Figure 4B shows that the proportion of ever-latent lineages in the replicating population corresponds to our phylogenetic results only when there is a large contribution of activated latent viruses every generation. For example, the population reaches >50% latently affected lineages in 10 years when *a*_*L*_ >10^−2^, *η* >10^−4^, and 1-ε_E_ = 0.01 with a reservoir half-life of 10 months.

When introducing drug treatment in the neutral model, with an efficacy at ε_E_ = ε_N_ = 0.85, the ever-latent fraction immediately increased to dominate the plasma virus population, regardless of *η*, *a*_*L*_, and *δ*_*L*_ values.

## Discussion and conclusions

We identified high proportions of lineages affected by latency in both the replicating plasma population and the latent reservoir in all patient types. Our findings suggest that HIV-1 cycles in and out of latent reservoirs regardless of patient treatment history. It is well known that among the few viruses that can be detected in patients under successful treatment, many have been reactivated from the latent pool [3,28,29]. Hence, we expected to find a notable proportion of lineages affected by latency in the plasma of treated patients; while this was true, however, the latency-affected proportion was also high in the plasma of untreated patients. This observation emphasizes that latency occurs regularly in the HIV-1 life cycle.

Our phylogenetic method shows that there are more lineages with reduced divergence in the reservoir than in the actively replicating population. This supports that we indeed find true latent lineages with our detection method. However, we did not find a perfect concordance in viruses from the plasma being non-latent and those from the reservoir being latent (virus with significant reduction in divergence). This is due to the fact that reservoir-derived viruses may have been deposited as recently as the day before sampling, while conversely, viruses in the plasma may have been activated recently. Therefore, binary classification based on where sequences come from (plasma vs. reservoir) is rather crude and ignores the history of the virus in either compartment, while our method gives a quantitative and continuous measure of latency periods regardless of compartment.

Similarly, although typically only small numbers of reactivated latent virus appear in the plasma population after substantial reductions in viremia due to effective antiviral therapy, this does not imply that only few viral lineages have cycled through the latent pool. Under neutrality, our dynamic model shows that even when the fraction of infected cells that become latent is small, and the activation rate of latently infected cells is low, the proportion of viral lineages that have ever been latent in the plasma is large. Analogously, a small stream may feed a large lake that also has a small outflow. If the lake was quickly evacuated, the small stream could not refill the lake immediately. Thus, the input has very little to do with the size of the holding tank (lake or viral load). We also show that introducing a constant fitness cost to ever-latent virus will make it much harder for such virus to survive. However, assuming a constant fitness cost on ever-latent viruses is not realistic for two main reasons: 1) ever-latent virus evolve, and can thus escape from immune pressure in time; 2) ever-latent virus may periodically have similar fitness as never-latent virus because the immune response evolves and changes over time. For example, when new antibody responses evolve, never-latent viruses will have reduced fitness until they acquire escape mutations. The ODE model we use here cannot include such effects.

Note that our phylogenetic detection method is a conservative estimate as we only compare taxa sampled at the same time, avoiding extrapolations between samplings, and likely underestimating the full impact of latency on branch lengths. Similarly, as rooting may affect latency detection in samples close to the root, we avoid potential errors in nearby samples by only considering each sampling time separately. Furthermore, the method considers relative rather than absolute latency; if latency is detected, it is with respect to at least one other sequence, which is by definition classified as non-latent. However, the reference taxa itself may be latent, in which case all relatively less divergent taxa must also have been latent. In fact, the prevalence of sequences from the latent reservoir with no significant divergence reduction (e.g., circle-2 in Figure 3) suggests that we may consistently underestimate latency. As the dynamic model considers absolute rather than relative latency the predicted fraction of ever-latent lineages is generally higher than identified in patient data by our phylogenetic detection method.

As latent lineages are detected relative to the reference taxa, it is crucial that the latter are placed correctly in the phylogeny to avoid overestimating latency due to artificially long reference taxa. In addition to hypermutation, which can elongate branches, within-patient recombination may also result in exaggerated branch lengths. A recombinant may be placed basal in a phylogeny with a short branch length, or it may sit near one of the parent strains, with mutations from the other parent rather than real substitutions significantly increasing the branch length. As detailed in Materials & Methods, we removed any reference taxa identified as potential recombinant or hypermutated taxa to prevent overestimating latency. To avoid effects of selection causing significant deviations in our latency detection, we analyzed 3^rd^ codon positions as most selection pressure is asserted on the protein level. While more sophisticated methods to analyze codons and codon positions are available [30-32], interestingly, however, we found that the identification of latent lineages was nearly the same when using all codon positions (compare Table 1 and Additional file 1: Table S1). This might be because immune selection is not strong enough to cause significantly different rates of evolution in coexisting lineages as all virus that survive to be detected have been under similar selection pressure, and over shorter time periods (<3 years) within-host evolution is consistent with neutrality [15]. Thus, the main contribution to HIV-1 rate differences stems from compartmentalized generation-time effects in latent reservoirs that slow down the evolution.

From the clinical perspective, latency is not a problem only relevant in patients with drug failure, but rather an integral part of the HIV-1 life cycle, expanding the evolutionary adaptation potential of the virus. Interestingly, Ho et al. recently showed that the latent reservoir may be up to 60-fold greater than previously estimated [33], further corroborating the central role of latency in infection dynamics. The recent findings suggesting preferential transmission of latent viruses, which necessitates a prevalence of latent lineages in the plasma, are also in line with our results [9-12]. From a biological perspective, latency explains the overdispersion in the molecular clock, previously observed but poorly understood. Thus, latency mechanistically explains evolutionary rate variation and is fundamental to understanding clinical outcomes related to pathogenesis and treatment.

## Methods

### Patient data

The representative data sets in Table 1, acquired from the Los Alamos HIV sequence database (http://www.hiv.lanl.gov/), consist of serially sampled sequence data from the *gag, pol*, or *env* genes of a total of 26 patients that were either untreated (*n* = 2) [18], successfully treated (*n* = 8) [17], had undergone treatment failure (*n* = 12) [19], or were elite suppressors (*n* = 4) [16]. The untreated patients were followed from primary HIV-1 infection (PHI), where the first sample was taken within a week of symptomatic PHI. These patients were still untreated at follow-up sampling 58—79 months later. Successfully treated patients consisted of infected adults with prolonged suppression of viremia to <50 copies/ml on a stable HAART regimen (mean duration of suppression prior to enrollment, 34 months: range, 11 to 79 months). For the patients with treatment failure, 11 of the 12 patients were on HAART at time of sampling, had been on current therapy for a mean of 9 months (0.5 to 20 months), and had been failing their current therapy for a mean of 7 months (0.5 to 20 months). All patients had a history of prior treatment failure with a mean time of 5 years of viral replication in the presence of antiretroviral therapy (range 8 to 128 months). Patient M-1 was not under treatment during sampling. The elite suppressors have maintained control of HIV-1 without HAART for several years, with mostly normal CD4+ T cell counts and plasma HIV-1 RNA levels below the limit of detection of clinical assays (<50 copies/ml). Additional file 1: Tables S1 and S2 present additional and detailed information about each patient and the longitudinal samples per patient.

We included patients with at least 10 sequences isolated from plasma and resting CD4 cells in the analysis. We did not distinguish whether sequences from resting cells were induced or replication-competent. Due to low viral loads, Bailey et al. [17] sampled the plasma of successfully treated patients every two to three days, with the samples generally consisting of only a few sequences. As HIV-1 sequences sampled two weeks apart or less show no significant evolution [15], we merged sequences sampled within one week into the same time point. The accession numbers for the data sets are AF349770-AF349906, GU993311-GU993518, DQ391282- DQ392955, and AY964187-AY964601, respectively.

### Phylogenetic reconstruction

We used the multiple sequence alignment software MAFFT version 7 [34] and GeneCutter (http://www.hiv.lanl.gov/) to create in-frame sequence alignments for each patient, allowing analyses of separate codon positions. Hypermutants were removed using the APOBEC context pattern test Hypermut 2.0 [35]. As each untreated patient was sampled shortly after PHI, we used the consensus sequence at the first time point, made by Consensus Maker v2.0.0 (http://www.hiv.lanl.gov/), as the reference sequence against which all sequences were compared to identify hypermutants. For all other patients, we used the full sequence alignments to make the respective consensus sequences.

Latency detection was first applied to the *N* taxa at the first time point, and the Phi-test was performed to identify potential recombination [36]. If φ ≥ 0.05, the set of sequences had no recombination signal, and the next time point was considered. If φ < 0.05, taxa were removed sequentially with replacement until the recombination signal in the alignment disappeared, i.e., each sequence was removed from the alignment one at a time, the Phi-test run, and the removed sequence replaced. If the φ-score become insignificant (φ ≥ 0.05) when a reference taxon was removed, it was removed from the original time-point alignment, and the latency test and the recombination identification procedure run again. If the recombination signal was lost with the removal of a non-reference taxa, the next time point was considered. If the recombination signal was not lost, the procedure was repeated for the alignment of *N* − 1 sequences with the highest φ-score. The latency detection and recombination identification tests were performed sequentially until φ ≥ 0.05, and no potentially recombinant reference taxa remained, before moving to the next time point. We developed the phylogenetic latency detection and recombination identification methods in R version 3.0.1 [37]. A similar algorithm using the Phi-test to identify/remove potential recombinants was previously developed by Salemi et al. [38].

After identifying and removing hypermutant and recombinant reference taxa, we inferred the phylogeny of each patient using PhyML 3.0 [39], with a general-time-reversible model that included rate variation among sites (*GTR* + Γ + *I*). As ancient viruses hiding in the latent reservoir can become activated late in the infection, rooting a phylogeny based on the sequences sampled at the first time point leads to the correct time structure only if the time point is early enough in the infection for latency to have had a negligible effect. As the untreated patients were sampled shortly after PHI, we used the most recent common ancestor (MRCA) of the sequences in the first time point to root each phylogeny. As the patients in the elite suppressor, treatment, and treatment failure categories were not sampled early in the infection, we rooted each phylogeny based on an outgroup (HIV-1 strain HXB2).

### Phylogenetic latency detection

HIV-1 lineages do not evolve independently; rather, they share much of their evolutionary history. To circumvent this dependency, we investigated all possible taxonomic sequence pairs, per time point, tracing their evolution back to the corresponding most recent common ancestor (pair-MRCA). In each such pair, the two resulting lineages will have independent mutational information, allowing us to calculate the Poisson probability *p* of accumulating the number of mutations observed on the shorter lineage *d*_*Short*_, given the evolutionary rate of the longer lineage *d*_*Long*_. Thus, we define latency when the shorter lineage indicated a $\Pr \left(\frac{\lambda^k}{k!}{e}^{-\lambda}\right)\le 0.05$, where *λ* = *d*_*Long*_ * *L* and *k* ≤ *d*_*Short*_ * *L* with sequence length *L*. For each latent taxon, we identified the corresponding reference taxon, defined as the longer lineage in the pair in which the latent signal was the strongest. Note that latent lineages can be identified in many pairwise comparisons. While this gives robustness to the test, the many tests performed also increases the risk of false positive test results. Thus, we controlled for the false discovery rate (FDR), informed by simulations as follows.

### Phylogenetic power and precision simulations

To understand the limitations of our method, we determined the sensitivity (correct identification of latent lineages; defined as true positives/[true positives + false positives] (TP/[TP + FP)) and specificity (correct identification of non-latent taxa; defined as true negatives/[true negatives + false negatives] (TN/[TN + FN])) of detecting latent taxa as a function of how much latency reduced a particular branch length in a phylogenetic tree, called *f*_*latent*_, and the original length of the latency affected branch. For this we simulated data by generating 90,000 random phylogenies consisting of 100 taxa each with an expected height of 0.1 substitutions/site and a sequence length of 1000 nt, reducing the length of a randomly chosen branch by a fraction *f*_*latent*_ = 0.1—0.9 of its original length. We also evaluated sensitivity and specificity as a function of divergence among lineages where latency had not affected the evolutionary rate by simulating 292,500 random trees at 0.001—0.3 substitutions/site tree height and reducing a random branch at *f*_*latent*_ = 0.1—0.9. Note that all of these simulations assumed a single latency period per tree.

Because we perform many tests, we also investigated by simulation the false discovery rate (FDR), defined as FP/[TP + FP]. To estimate the FDR, we varied the number of taxa in a sample (range 10—60), *f*_latent_ (range 0.5—0.9), and the number of latent periods (range 1—10). At each parameter combination we simulated 10,000 random trees, totaling 3,000,000 simulations, to estimate the FDR relevant to our testing conditions. In addition, we simulated the FDR on 50,000 trees for each patient sample (1—18 time points/patient), adjusting for the precise number of taxa and estimated minimum number of independent latency periods, totaling 7,800,000 patient specific simulations (Additional file 1: Table S2).

### Dynamic model

To examine the impact of latency on the overall evolution of HIV-1 within a patient, we extended a basic model of HIV-1 latency developed by Rong et al. [26,27] to explicitly account for never-latent and ever-latent virus lineages. Ever-latent lineages consist of viruses that have cycled through a latent reservoir at some time in their past, while those classified as never-latent have always been actively replicating. The model is described by the following set of ordinary differential equations (ODEs):

$$ \begin{array}{l}\frac{dT}{dt}=\lambda -{\delta}_TT-\left(1-{\varepsilon}_E\right)kT{V}_E-\left(1-{\varepsilon}_N\right)kT{V}_N\\ {}\frac{d{I}_N}{dt}=\left(1-{\varepsilon}_N\right)\left(1-\eta \right)kT{V}_N-{\delta}_I{I}_N\\ {}\frac{d{V}_N}{dt}=N{\delta}_I{I}_N-c{V}_N\\ {}\frac{d{I}_E}{dt}=\left(1-{\varepsilon}_E\right)\left(1-\eta \right)kT{V}_E-{\delta}_I{I}_E+{a}_LL\\ {}\frac{d{V}_E}{dt}=N{\delta}_I{I}_N-c{V}_E\\ {}\frac{dL}{dt}=\left(1-{\varepsilon}_E\right)\eta kT{V}_E+\left(1-{\varepsilon}_N\right)\eta kT{V}_N-{a}_LL-{\delta}_L\end{array} $$

where *T* are uninfected CD4+ T cells , *V*_*N*_ and *V*_*E*_ the never-latent and ever-latent virus concentrations in plasma, respectively, *I*_*N*_ and *I*_*E*_ cells infected with never-latent and ever-latent virus, and *L* are resting latently infected cells.

Uninfected cells proliferate at rate *λ,* are infected according to the laws of mass action at rate *k*, and die at rate *δ*_*T*_, while infected cells die at rate *δ*_*I*_. The fraction of infections resulting in latency is *η*, while latent cells are activated at rate *a*_*L*_ to become productively infected cells. Infected cells have a burst size of *N* and plasma virus decay at rate *c*. The fitness effect of the immune response reduces infection by a fraction of *ε*_*N*_ for never-latent lineages and *ε*_*E*_ for ever-latent lineages. To investigate the effects of anti-viral drugs we set *ε*_*E*_ *= ε*_*N*_ = 0.85. We did not explicitly model the regeneration of latently infected cells [26,27], but rather calibrated the net growth rate *δ*_*L*_ of the latent cells to achieve a desired half life *H*_*life*_ of the latent reservoir for a given activation rate, namely, *δ*_*L*_ *= ln(2)/H*_*life*_*-a*_*L*_. As the half life has been estimated to range between 6 months [40,41] and 44 months [42,43], we chose a broad range between 1 and 100 months.

The other parameters and variables used in the model are described in Table S2. As in [27], we set the initial conditions for uninfected CD4 T cells, *T,* and never-latent virus, *V*_*N*_, to be 10^6^ cells/ml [27] and 50 RNA copies/ml, respectively. In addition we assumed that there were no productively infected cells, resting latently infected cells, or virus from ever-latent lineages at *t* = 0. The model was solved numerically using the R package deSolve [44].

## Additional file

Additional file 1: Figure S1. 26 individual patient trees. **Figure S2.** Raw sensitivity simulation results. **Table S1.** Patient data and results based on all codon positions. **Table S2.** Detailed results based on 3^rd^ codon positions. **Table S3.** Dynamic model parameters and variables.
