# Supplementary material for: Reduced evolutionary rates in HIV-1 reveal extensive latency periods among replicating lineages
Source: Retrovirology. 2014 Oct 16;11:81. doi: 10.1186/s12977-014-0081-0 (PMC4201670; doi:10.1186/s12977-014-0081-0)
Supplement: Additional file 1: Figure S1. — 26 individual patient trees. Figure S2. Raw sensitivity simulation results. Table S1. Patient data and results based on all codon positions. Table S2. Detailed results based on 3rd codon positions. Table S3. Dynamic model parameters and variables. [file 12977_2014_81_MOESM1_ESM.docx]

**Reduced evolutionary rates in HIV-1 reveal extensive latency periods among replicating lineages**

Taina T. Immonen & Thomas Leitner

Theoretical Biology & Biophysics, Los Alamos National Laboratory, Los Alamos, NM 87545, USA

**SUPPLEMENTAL MATERIALS**

****Figure S1. Individual patient phylogenies based on 3^rd^ codon positions.** Symbols and colors as described in Figure 3 caption of the main article.


**************************************************


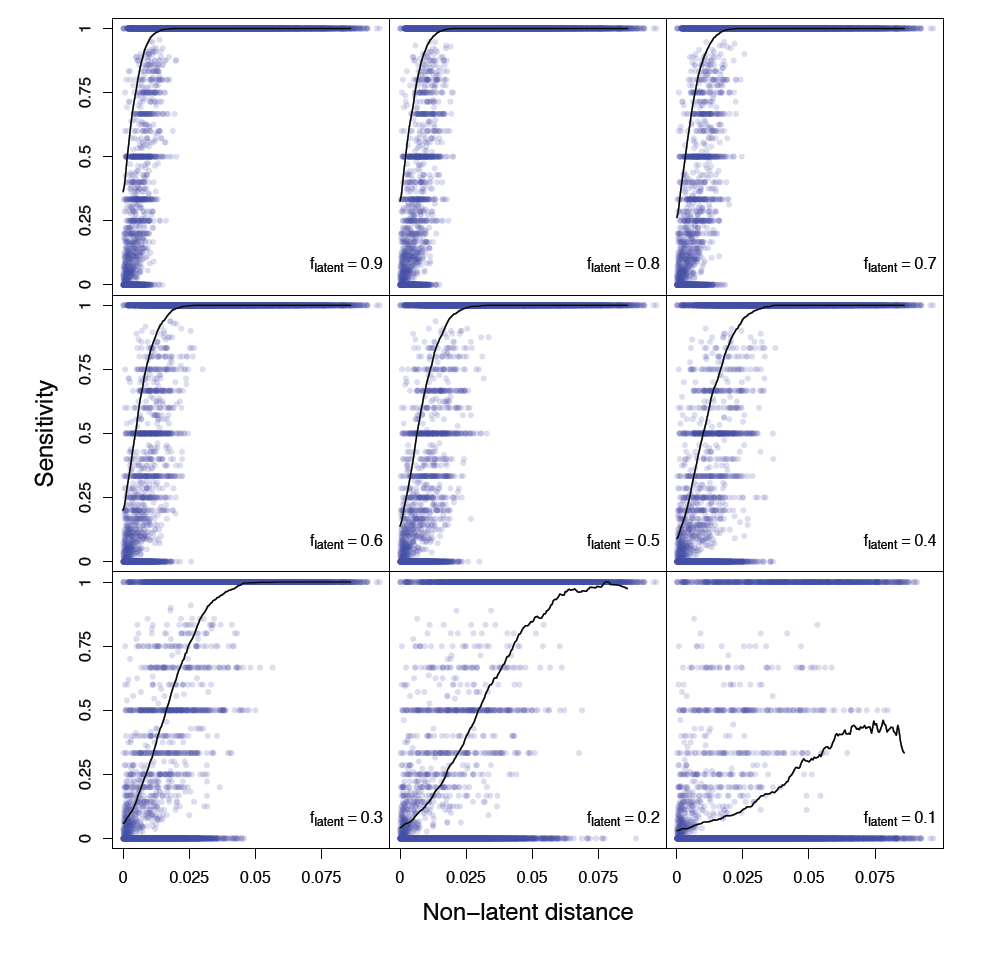


**Figure S2. Raw results from sensitivity and specificity simulations**. Trees with 100 taxa and one random internal or external branch affected by latency at *f*_latent_ = 0.9–0.1 of the corresponding non-latent genetic distance (10,000 simulations/*f*_latent_). Each blue point represents one tree simulation, where deeper color shows overlapping points, and black lines show moving average for general trends.

**Table S1. Summary of patient data and latency results based on all codon positions.**

| **Patient** | **Patient** | **Gene** | **L** | **Time** | **No.** | **H** | **R** | **No. (%) latent taxa detected** | | | |
| --- | --- | --- | --- | --- | --- | --- | --- | --- | --- | --- | --- |
| **ID** | **type** |  |  | **points** | **taxa** |  |  | **total** | **plasma** | **reservoir** | **FP** |
| K-B | U | *env* | 714 | 3 | 69 | 1 | 2 | 38 (79.2) | 20 (71.4) | 18 (90.0) | 1.03 |
| K-G | U | *env* | 729 | 3 | 68 | 0 | 5 | 30 (65.2) | 15 (57.7) | 15 (75.0) | 0.32 |
| O-1 | E | *gag* | 1386 | 3 | 23 | 0 | 0 | 15 (75.0) | 1 (20.0) | 14 (93.3) | 0.84 |
| O-7 | E | *gag* | 1271 | 9 | 62 | 1 | 0 | 28 (50.0) | 21 (44.7) | 7 (77.8) | 0.50 |
| O-8 | E | *gag* | 1298 | 8 | 122 | 0 | 0 | 76 (65.5) | 30 (43.4) | 46 (97.9) | 4.16 |
| O-9 | E | *gag* | 1271 | 5 | 57 | 2 | 0 | 34 (68.0) | 5 (25.0) | 29 (96.7) | 0.64 |
| M-1 | F | *pol* | 985 | 1 | 22 | 0 | 0 | 10 (45.5) | 4 (40.0) | 6 (50.0) | 0.03 |
| M-96 | F | *pol* | 637 | 2 | 19 | 0 | 0 | 6 (31.6) | 0 (0.0) | 6 (50.0) | 0.31 |
| M-100 | F | *pol* | 637 | 1 | 16 | 0 | 0 | 13 (81.2) | 3 (60.0) | 10 (90.9) | 0.02 |
| M-102 | F | *pol* | 637 | 3 | 109 | 0 | 1 | 83 (76.9) | 25 (89.3) | 58 (72.5) | 3.62 |
| M-105 | F | *pol* | 637 | 3 | 55 | 0 | 1 | 41 (75.9) | 19 (65.6) | 22 (88.0) | 0.09 |
| M-110 | F | *pol* | 637 | 1 | 12 | 0 | 0 | 11 (91.7) | 5 (83.3) | 6 (100) | 0.08 |
| M-112 | F | *pol* | 637 | 1 | 25 | 0 | 3 | 5 (22.7) | 2 (22.2) | 3 (23.1) | 0.77 |
| M-122 | F | *pol* | 637 | 1 | 14 | 0 | 0 | 8 (57.1) | 1 (16.7) | 7 (87.5) | 0.22 |
| M-126 | F | *pol* | 637 | 1 | 18 | 0 | 0 | 14 (77.8) | 4 (66.7) | 10 (83.3) | 0.47 |
| M-127 | F | *pol* | 985 | 1 | 14 | 0 | 0 | 6 (42.7) | 3 (75.0) | 3 (30.0) | 0.06 |
| M-137 | F | *pol* | 637 | 2 | 33 | 0 | 0 | 18 (54.5) | 1 (6.3) | 17 (100) | 3.86 |
| M-138 | F | *pol* | 985 | 1 | 11 | 0 | 0 | 3 (27.2) | 0 (0.0) | 3 (60.0) | 0.02 |
| B-113 | T | *pol* | 546 | 10 | 51 | 0 | 0 | 23 (48.9) | 6 (26.1) | 17 (70.8) | 0.06 |
| B-134 | T | *pol* | 546 | 5 | 81 | 0 | 1 | 38 (50.7) | 2 (28.6) | 36 (52.9) | 0.63 |
| B-136 | T | *pol* | 546 | 12 | 118 | 0 | 0 | 37 (32.5) | 5 (10.4) | 32 (48.5) | 19.4 |
| B-139 | T | *pol* | 546 | 10 | 141 | 0 | 1 | 98 (78.4) | 26 (60.5) | 72 (87.8) | 1.21 |
| B-147 | T | *pol* | 546 | 10 | 136 | 0 | 3 | 107 (82.9) | 25 (58.1) | 82 (95.3) | 3.66 |
| B-148 | T | *pol* | 546 | 4 | 193 | 0 | 0 | 144 (94.7) | 12 (85.7) | 132 (95.7) | 5.64 |
| B-154 | T | *pol* | 546 | 18 | 454 | 0 | 15 | 208 (63.6) | 111 (51.4) | 97 (91.5) | 10.6 |
| B-154 | T | *env* | 306 | 3 | 67 | 3 | 0 | 33 (51.6) | 15 (35.7) | 18 (81.8) | 1.13 |

Footnotes: Patient type, U = untreated, E = elite controller, F = failed treatment, T = successful treatment; L = original sequence length of all codon positions; H = number of hypermutants removed; R = number of recombinants removed; No. taxa = number of taxa after H and R removal; FP = estimated number of false positives.

**Table S2. Detailed patient information and latency detection results based on 3^rd^ codon positions.**

| **Patient** | **Time** | **No. taxa** | | | **No. latent taxa detected** | | | **Latent** | **Mean** | **FDR** |
| --- | --- | --- | --- | --- | --- | --- | --- | --- | --- | --- |
| **ID** | **(week)** | **total** | **plasma** | **reservoir** | **total** | **plasma** | **reservoir** | **periods** | **height** |  |
| **K-B** | 292 | 7 | 7 | 0 | 5 | 5 | . | 2 | 9.0e-02 | 6.7e-03 |
|  | 316 | 43 | 22 | 21 | 40 | 19 | 21 | 15 | 1.5e-01 | 1.6e-02 |
| **K-G** | 232 | 9 | 9 | 0 | 7 | 7 | . | 2 | 4.5e-02 | 3.2e-02 |
|  | 276 | 42 | 22 | 20 | 39 | 19 | 20 | 14 | 6.6e-02 | 1.8e-02 |
| **O-1** | 0 | 9 | 0 | 9 | 8 | . | 8 | 1 | 1.1e-02 | 7.6e-02 |
|  | 44 | 3 | 3 | 0 | 2 | 2 | . | 2 | 6.4e-02 | 0 |
|  | 160 | 8 | 2 | 6 | 6 | 0 | 6 | 1 | 3.7e-02 | 1.9e-02 |
| **O-7** | 0 | 17 | 17 | 0 | 14 | 14 | 0 | 6 | 3.4e-02 | 7.0e-03 |
|  | 16 | 2 | 0 | 2 | 1 | 0 | 1 | 1 | 3.6e-02 | 0 |
|  | 32 | 4 | 0 | 4 | 0 | . | 0 | 0 | 8.0e-03 | 0 |
|  | 40 | 6 | 6 | 0 | 5 | 5 | . | 4 | 3.8e-02 | 9.6e-04 |
|  | 104 | 3 | 3 | 0 | 0 | 0 | . | 0 | 3.0e-03 | 0 |
|  | 160 | 5 | 5 | 0 | 3 | 3 | . | 1 | 3.2e-02 | 1.7e-02 |
|  | 188 | 4 | 4 | 0 | 0 | 0 | . | 0 | 3.4e-03 | 0 |
|  | 200 | 2 | 2 | 0 | 1 | 1 | . | 1 | 1.6e-02 | 0 |
|  | 236 | 10 | 10 | 0 | 3 | 3 | . | 1 | 2.9e-03 | 5.6e-02 |
| **O-8** | 32 | 15 | 3 | 12 | 11 | 0 | 11 | 5 | 2.0e-02 | 2.8e-03 |
|  | 188 | 5 | 5 | 0 | 1 | 1 | . | 1 | 4.9e-03 | 0 |
|  | 204 | 7 | 7 | 0 | 0 | 0 | . | 0 | 5.2e-03 | 0 |
|  | 212 | 12 | 12 | 0 | 0 | 0 | . | 0 | 5.4e-03 | 0 |
|  | 240 | 8 | 8 | 0 | 5 | 5 | . | 2 | 1.3e-02 | 6.5e-03 |
|  | 244 | 8 | 8 | 0 | 7 | 7 | . | 4 | 2.9e-02 | 1.8e-03 |
|  | 248 | 46 | 11 | 35 | 36 | 2 | 34 | 3 | 2.4e-02 | 1.0e-01 |
| **O-9** | 0 | 5 | 5 | 0 | 0 | 0 | . | 0 | 6.8e-02 | 0 |
|  | 24 | 18 | 2 | 16 | 17 | 1 | 16 | 7 | 7.2e-02 | 3.4e-03 |
|  | 40 | 3 | 3 | 0 | 2 | 2 | . | 2 | 1.7e-02 | 0 |
|  | 204 | 14 | 8 | 6 | 6 | 0 | 6 | 2 | 3.1e-02 | 2.9e-02 |
|  | 252 | 10 | 2 | 8 | 7 | 0 | 7 | 2 | 3.1e-02 | 1.1e-02 |
| **M-1** | 0 | 22 | 10 | 12 | 12 | 6 | 6 | 9 | 2.2e-02 | 3.4e-03 |
| **M-96** | 0 | 3 | 3 | 0 | 0 | 0 | . | 0 | 1.6e-03 | 0 |
|  | 12 | 16 | 4 | 12 | 0 | 0 | 0 | 0 | 1.6e-02 | 0 |
| **M-100** | 0 | 16 | 5 | 11 | 4 | 2 | 2 | 3 | 2.8e-02 | 1.7e-02 |
| **M-102** | 0 | 47 | 9 | 38 | 31 | 2 | 29 | 15 | 8.5e-02 | 6.4e-03 |
|  | 12 | 38 | 8 | 30 | 20 | 5 | 15 | 14 | 5.9e-02 | 4.6e-03 |
|  | 24 | 23 | 11 | 12 | 8 | 5 | 3 | 5 | 4.6e-02 | 1.9e-02 |
| **M-105** | 0 | 17 | 9 | 8 | 14 | 6 | 8 | 6 | 1.5e-01 | 7.0e-03 |
|  | 12 | 19 | 8 | 11 | 16 | 6 | 10 | 13 | 2.0e-01 | 3.3e-05 |
|  | 24 | 19 | 12 | 7 | 18 | 12 | 6 | 12 | 2.4e-01 | 0 |
| **M-110** | 0 | 12 | 6 | 6 | 11 | 5 | 6 | 5 | 8.0e-02 | 6.0e-03 |
| **M-112** | 0 | 25 | 9 | 16 | 18 | 5 | 13 | 8 | 3.1e-02 | 4.6e-03 |
| **M-122** | 0 | 14 | 6 | 8 | 7 | 0 | 7 | 1 | 1.8e-02 | 4.8e-02 |
| **M-126** | 0 | 18 | 6 | 12 | 5 | 4 | 1 | 3 | 2.4e-02 | 2.6e-02 |
| **M-127** | 0 | 14 | 4 | 10 | 8 | 4 | 4 | 6 | 6.9e-02 | 3.6e-03 |
| **M-137** | 0 | 5 | 5 | 0 | 1 | 1 | . | 1 | 3.3e-02 | 0 |
|  | 4 | 28 | 11 | 17 | 16 | 0 | 16 | 3 | 2.6e-02 | 3.9e-03 |
| **M-138** | 0 | 11 | 6 | 5 | 0 | 0 | 0 | 0 | 4.1e-02 | 0 |
| **B-113** | 0 | 27 | 3 | 24 | 25 | 3 | 22 | 10 | 9.5e-02 | 2.6e-03 |
|  | 4 | 2 | 2 | 0 | 1 | 1 | . | 1 | 4.3e-02 | 0 |
|  | 5 | 3 | 3 | 0 | 2 | 2 | . | 1 | 3.4e-02 | 0 |
|  | 6 | 2 | 2 | 0 | 1 | 1 | . | 1 | 6.2e-02 | 0 |
|  | 7 | 2 | 2 | 0 | 1 | 1 | . | 1 | 3.4e-02 | 0 |
|  | 8 | 3 | 3 | 0 | 0 | 0 | . | 0 | 1.2e-02 | 0 |
|  | 10 | 2 | 2 | 0 | 1 | 1 | . | 1 | 4.3e-02 | 0 |
|  | 13 | 2 | 2 | 0 | 1 | 1 | . | 1 | 6.2e-02 | 0 |
|  | 14 | 2 | 2 | 0 | 0 | 0 | . | 0 | 1.7e-02 | 0 |
|  | 15 | 2 | 2 | 0 | 1 | 1 | . | 1 | 3.6e-02 | 0 |
| **B-134** | 0 | 28 | 0 | 28 | 22 | 0 | 22 | 11 | 5.2e-02 | 9.9e-04 |
|  | 5 | 3 | 3 | 0 | 1 | 1 | 0 | 1 | 3.7e-02 | 0 |
|  | 12 | 2 | 2 | 0 | 0 | 0 | 0 | 0 | 2.3e-02 | 0 |
|  | 15 | 2 | 2 | 0 | 0 | 0 | 0 | 0 | 2.0e-02 | 0 |
|  | 30 | 41 | 0 | 41 | 31 | 0 | 31 | 7 | 5.4e-02 | 3.8e-02 |
| **B-136** | 0 | 4 | 4 | 0 | 0 | 0 | . | 0 | 2.8e-03 | 0 |
|  | 4 | 6 | 6 | 0 | 1 | 1 | . | 1 | 1.6e-02 | 3.1e-05 |
|  | 5 | 3 | 3 | 0 | 0 | 0 | . | 0 | 9.5e-03 | 0 |
|  | 7 | 2 | 2 | 0 | 0 | 0 | . | 0 | 5.7e-03 | 0 |
|  | 11 | 10 | 10 | 0 | 0 | 0 | . | 0 | 2.8e-03 | 0 |
|  | 12 | 8 | 8 | 0 | 0 | 0 | . | 0 | 7.1e-03 | 0 |
|  | 13 | 3 | 3 | 0 | 1 | 1 | . | 1 | 2.5e-02 | 0 |
|  | 14 | 4 | 4 | 0 | 1 | 1 | . | 1 | 1.5e-02 | 0 |
|  | 25 | 67 | 1 | 66 | 46 | 1 | 45 | 5 | 2.3e-02 | 3.0e-01 |
|  | 86 | 3 | 3 | 0 | 1 | 1 | . | 1 | 1.7e-02 | 0 |
| **B-139** | 0 | 37 | 3 | 34 | 32 | 0 | 32 | 7 | 9.9e-02 | 9.2e-02 |
|  | 4 | 9 | 9 | 0 | 1 | 1 | . | 1 | 6.6e-02 | 1.5e-03 |
|  | 5 | 4 | 4 | 0 | 2 | 2 | . | 2 | 3.2e-02 | 5.0e-02 |
|  | 9 | 4 | 4 | 0 | 1 | 1 | . | 1 | 3.2e-02 | 0 |
|  | 12 | 8 | 8 | 0 | 3 | 3 | . | 2 | 1.1e-01 | 1.2e-02 |
|  | 13 | 3 | 3 | 0 | 1 | 1 | . | 1 | 3.2e-02 | 0 |
|  | 14 | 2 | 2 | 0 | 1 | 1 | . | 1 | 3.0e-02 | 0 |
|  | 15 | 2 | 2 | 0 | 1 | 1 | . | 1 | 9.1e-02 | 0 |
|  | 30 | 26 | 0 | 26 | 18 | . | 18 | 5 | 5.8e-02 | 3.9e-02 |
|  | 75 | 31 | 8 | 23 | 28 | 8 | 20 | 8 | 1.0e-01 | 4.3e-03 |
| **B-147** | 0 | 36 | 9 | 27 | 34 | 9 | 25 | 13 | 1.5e-01 | 5.0e-03 |
|  | 5 | 3 | 3 | 0 | 2 | 2 | . | 1 | 3.2e-02 | 0 |
|  | 6 | 2 | 2 | 0 | 1 | 1 | . | 1 | 2.5e-02 | 0 |
|  | 8 | 7 | 7 | 0 | 6 | 6 | . | 4 | 1.8e-01 | 2.0e-03 |
|  | 9 | 2 | 2 | 0 | 1 | 1 | . | 1 | 4.4e-02 | 0 |
|  | 10 | 7 | 7 | 0 | 2 | 2 | . | 1 | 2.3e-02 | 5.5e-04 |
|  | 12 | 3 | 3 | 0 | 2 | 2 | . | 1 | 1.3e-01 | 0 |
|  | 14 | 4 | 4 | 0 | 3 | 3 | . | 2 | 6.6e-02 | 0 |
|  | 15 | 2 | 2 | 0 | 1 | 1 | . | 1 | 5.9e-02 | 0 |
|  | 28 | 66 | 4 | 62 | 60 | 1 | 59 | 16 | 1.8e-01 | 3.7e-02 |
| **B-148** | 0 | 43 | 5 | 38 | 38 | 4 | 34 | 14 | 9.3e-02 | 2.6e-02 |
|  | 12 | 6 | 6 | 0 | 0 | 0 | . | 0 | 4.6e-02 | 0 |
|  | 28 | 49 | 1 | 48 | 42 | 1 | 41 | 14 | 9.0e-02 | 2.1e-02 |
|  | 50 | 54 | 2 | 52 | 49 | 2 | 47 | 12 | 7.7e-02 | 4.3e-02 |
| **B-154** | 0 | 31 | 6 | 25 | 23 | 4 | 19 | 13 | 9.6e-02 | 3.6e-03 |
|  | 4 | 14 | 14 | 0 | 3 | 3 | . | 2 | 6.8e-02 | 3.0e-02 |
|  | 5 | 8 | 8 | 0 | 7 | 7 | . | 2 | 8.0e-02 | 3.1e-04 |
|  | 6 | 13 | 13 | 0 | 6 | 6 | . | 4 | 9.4e-02 | 6.5e-03 |
|  | 7 | 10 | 10 | 0 | 7 | 7 | . | 2 | 4.5e-02 | 1.1e-02 |
|  | 8 | 11 | 11 | 0 | 0 | 0 | . | 0 | 3.4e-02 | 0 |
|  | 9 | 18 | 18 | 0 | 1 | 1 | . | 1 | 3.6e-02 | 1.3e-02 |
|  | 10 | 16 | 16 | 0 | 0 | 0 | . | 0 | 5.9e-02 | 0 |
|  | 11 | 17 | 17 | 0 | 0 | 0 | . | 0 | 1.0e-03 | 0 |
|  | 13 | 13 | 13 | 0 | 0 | 0 | . | 0 | 3.4e-02 | 0 |
|  | 14 | 16 | 16 | 0 | 1 | 1 | . | 1 | 3.6e-02 | 9.0e-03 |
|  | 15 | 22 | 22 | 0 | 1 | 1 | . | 1 | 5.9e-02 | 2.3e-02 |
|  | 30 | 47 | 9 | 38 | 29 | 8 | 21 | 14 | 9.2e-02 | 2.4e-02 |
|  | 58 | 45 | 23 | 22 | 36 | 23 | 13 | 9 | 1.0e-01 | 7.4e-02 |
|  | 65 | 31 | 8 | 23 | 23 | 8 | 15 | 10 | 1.0e-01 | 5.8e-03 |
|  | 71 | 12 | 4 | 8 | 9 | 4 | 5 | 5 | 1.2e-01 | 3.8e-03 |
|  | 83 | 5 | 5 | 0 | 4 | 4 | . | 2 | 1.1e-01 | 4.1e-03 |
|  | 91 | 6 | 6 | 0 | 0 | 0 | . | 0 | 4.2e-02 | 0 |
| **B-154** | 0 | 15 | 15 | 0 | 4 | 4 | . | 3 | 1.0e-01 | 1.4e-02 |
|  | 44 | 40 | 18 | 22 | 38 | 18 | 20 | 9 | 1.8e-01 | 1.1e-02 |
|  | 91 | 9 | 9 | 0 | 8 | 8 | . | 1 | 1.1e-01 | 7.6e-02 |

Footnotes:

*Time (week)* is in relation to first sample, for K-B and K-G time is relative to PHI.

*Latent periods* refers to the minimum number of independent latency periods that must have occurred to explain the phylogenetic clustering.

*Mean height* is the mean height of non-latent taxa from the MRCA of all taxa sampled at one time point measured in substitutions/site.

*FDR* is the estimated false discovery rate in 50,000 phylogenies with the same number of taxa and latent periods as in the corresponding patient sample, with *f*_latent_ = 0.5—0.9.

**Table S3. Dynamic model parameters and variables**


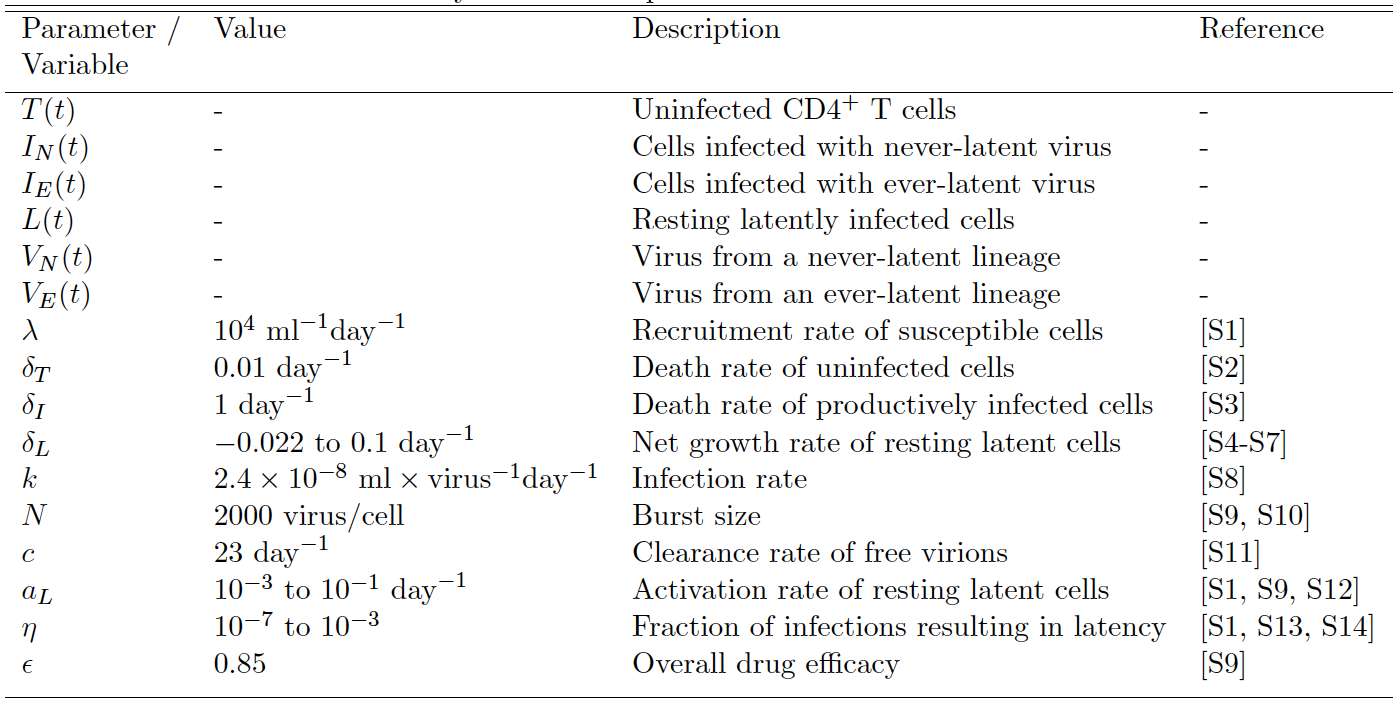


Table S3 References:

[S1] Callaway, D. S., and Perelson, A. S. (2002). HIV-1 infection and low steady state viral loads. Bulletin of Mathematical Biology, 64(1), 29-64

[S2] Mohri, H., Bonhoeffer, S., Monard, S., Perelson, A.S., and Ho, D.D. (1998). Rapid turnover of T l ymphocytes in SIV-infected rhesus macaques. Science, 279(5354), 1223-1227.

[S3] Markowitz M, Louie M, Hurley A, Sun E, Di Mascio M, et al. (2003). A novel antiviral intervention results in more accurate assessment of human immunodeficiency virus type 1 replication dynamics and T-cell decay in vivo. Journal of virology, 77(8), 5037-5038.

[S4] Ramratnam B, Mittler JE, Zhang L, Boden D, Hurley A, et al. (2000) The decay of the latent reservoir of replication-competent HIV-1 is inversely correlated with the extent of residual viral replication during prolonged anti-retroviral therapy. Nat Med 6: 82–85.

[S5] Zhang L, Ramratnam B, Tenner-Racz K, He Y, Vesanen M, et al. (1999) Quantifying residual HIV-1 replication in patients receiving combination antiretroviral therapy. N Engl J Med 340: 1605–1613.

[S6] Finzi D, Blankson J, Siliciano JD, Margolick JB, Chadwick K, et al. (1999) Latent infection of CD4+ T cells provides a mechanism for lifelong persistence of HIV-1, even in patients on effective combination therapy. Nat Med 5: 512–517.

[S7] Siliciano JD, Kajdas J, Finzi D, Quinn TC, Chadwick K, et al. (2003) Long-term follow-up studies confirm the stability of the latent reservoir for HIV-1 in resting CD4+ T cells. Nat Med 9: 727–728.

[S8] Perelson, A.S, Kirschner, D.E., De Boer, R. (1993). Dynamics of HIV infection of CD4+ T cells. Mathematical Biosciences, 114(1), 81-125

[S9] Rong L., Perelson A.S. (2009). Modeling Latently Infected Cell Activation: Viral and Latent Reservoir Persistence, and Viral Blips in HIV-infected Patients on Potent Therapy. PLoS Comput Biol 5(10): e1000533.

[S10] Hockett, R.D., Kilby, J. M., Derdeyn, C A., Saag, M.S., Sillers, M., et al. (1999). Constant mean viral copy number per infected cell in tissues regardless of high, low, or undetectable plasma HIV RNA. The Journal of experimental medicine, 189(10), 1545-1554.

[S11] Ramratnam, B., Bonhoeffer, S., Binley, J., Hurley, A., Zhang, L., et al. (1999). Rapid production and clearance of HIV-1 and hepatitis C virus assessed by large volume plasma apheresis. The Lancet, 354(9192), 1782-1785.

[S12] Rong L, Perelson AS (2009) Asymmetric division of activated latently infected cells may explain the decay kinetics of the HIV-1 latent reservoir and intermittent viral blips. Math Biosci 217: 77–87

[S13] Jones, L. E., and Perelson, A. S. (2007). Transient viremia, plasma viral load, and reservoir replenishment in HIV-infected patients on antiretroviral therapy. Journal of acquired immune deficiency syndromes, 45(5), 483.

[S14] Strain MC, Little SJ, Daar ES, Havlir DV, Gunthard HF, et al. (2005) Effect of treatment, during primary infection, on establishment and clearance of cellular reservoirs of HIV-1. J Infect Dis 191: 1410–1418.
